# Nursing as a player in tackling vaccine hesitancy and refusal

**DOI:** 10.1590/0034-7167.202477suppl101

**Published:** 2024-03-08

**Authors:** Mônica Lá-Salette da Costa Godinho, Simone Albino da Silva, Gisele Acerra Biondo Pietrafesa

**Affiliations:** I Universidade Federal de Alfenas. Alfenas. Minas Gerais. Brazil

Since the 1970s, the Brazilian Ministry of Health established the Brazilian National Immunization Program (PNI - Programa Nacional de Imunização), which preceded the Brazilian Health System and which was incorporated and strengthened due to the decentralized model to municipalities, but under single command at central level. Its objective was and still is to coordinate vaccination actions to control, eradicate and eliminate vaccinepreventable diseases.

In the 50 years of the program’s existence, in addition to offering free coverage to all Brazilians, PNI actions have usually been increased over time, diversifying the variety of immunobiological agents implemented in routine vaccination calendars or offered as special immunobiological agents as well as the number of vaccines offered to the population, thus becoming one of the largest public vaccination programs in the world.

Thus, the PNI collaborated in the prevention of several diseases, with significant drops in incidence rates of vaccine-preventable diseases and improvements in health indicators, promoting a positive impact on the Brazilian population’s life expectancy^(1)^. On the other hand, the decrease in the incidence of some diseases has led to a false idea of their disappearance, generating low adherence to vaccination and reducing the importance given to vaccines as the main means of prevention.

In a study published in 2020, the Brazilian National PNI Surveillance System identified a decrease in vaccination coverage since 2010 and heterogeneity between municipalities, resulting in the reintroduction of controlled diseases, such as measles and polio^(2)^.

This context, accentuated by the social isolation generated by the coronavirus pandemic, with the aggravating and constant dissemination of fake news caused by media expansion, particularly with the presence of the internet and its high propagation power^(3)^, added to the population’s lack of knowledge on the subject, contributed to behaviors such as vaccine hesitancy and refusal, with a very negative drop in vaccination coverage. Internet materials show content in which doubts are placed alongside emotional appeals, mixed with real news among fake news, which ends up generating credibility to the misinformation conveyed^(4)^.

Nursing, which has contributed to the effectiveness of PNI over time, undertaking its technical, scientific and ethical skills in its development, is faced with this new challenge: vaccine hesitancy and refusal resulting from untruths that attempt to deconstruct scientific knowledge based on evidence and scientific methods.

Considering the leading role of nursing in PNI, care must be taken to base its work on the best and most recent scientific evidence, considering the constant evolution of knowledge and being able to promote health education inside and outside Basic Health Units, appropriating and disseminating information about vaccination with a scientific basis and adapted to society’s level of understanding.

The trained and qualified nursing team is responsible for the procedures in vaccination rooms, including handling, conservation, preparation, administration, recording and disposal of waste resulting from vaccination actions, as well as team continuing education, paying attention to the population’s vaccination coverage and occurrence of adverse events.

Immunization management and coordination in states and municipalities must guarantee training on immunization and the cold chain and, thus, carry out immunization in a resolute and quality manner so that high and homogeneous vaccination coverage can be achieved in order to maintain vaccine-preventable disease elimination or control.

It should be noted that, throughout the PNI chain of actions, the work of the nursing team under the leadership of nurses is strategic, from central level planning to the leadership of local teams that work in vaccination rooms.

In the meantime, it is important to highlight the need for more nursing research to be carried out to understand the impact of false news disseminated among the population related to vaccine hesitancy and refusal and to develop effective communication strategies with society to reverse low vaccination coverage and rebuild the Brazilian population’s trust in PNI.
